# Non‐Response to Obeticholic Acid Is Associated With Heightened Risks of Developing Clinical Events in Primary Biliary Cholangitis

**DOI:** 10.1111/apt.70378

**Published:** 2025-09-22

**Authors:** Nadir Abbas, Rachel Smith, Ellina Lytvyak, Miki Scaravaglio, Neil Halliday, Amal Almahroos, Nadia Eden, Diane Lloyd‐Madden, Sanchit Sharma, James Ferguson, Jessica K. Dyson, Douglas Thorburn, David Jones, Aldo J. Montano‐Loza, Marco Carbone, Pietro Invernizzi, George Mells, Emma L. Culver, Palak J. Trivedi

**Affiliations:** ^1^ National Institute for Health and Care Research (NIHR) Birmingham Biomedical Research Centre Centre for Liver and Gastrointestinal Research, University of Birmingham UK; ^2^ Liver Unit, University Hospitals Birmingham Queen Elizabeth Birmingham UK; ^3^ Institute of Immunology, Immunity and Immunotherapy University of Birmingham UK; ^4^ Academic Department of Medical Genetics University of Cambridge Cambridge UK; ^5^ Division of Gastroenterology and Liver Unit University of Alberta Edmonton Canada; ^6^ Department of Medicine and Surgery University of Milano‐Bicocca Milan Italy; ^7^ Institute of Liver and Digestive Health, University College London London UK; ^8^ Sheila Sherlock Liver Centre, Royal Free London NHS Foundation Trust London UK; ^9^ Department of Hepatology Newcastle Upon Tyne Hospital NHS Foundation Trust Newcastle UK; ^10^ NIHR Newcastle Biomedical Research Centre Newcastle University Newcastle UK; ^11^ Division of Gastroenterology Center for Autoimmune Liver Diseases, European Reference Network on Hepatological Diseases (ERN RARE‐LIVER), IRCCS Fondazione San Gerardo Dei Tintori Monza Italy; ^12^ Translational Gastroenterology and Liver Unit, John Radcliffe Hospital, Nuffield Department of Medicine University of Oxford Oxford UK

**Keywords:** bezafibrate, cirrhosis, fibric acid, obeticholic acid

## Abstract

**Objective:**

Biochemical non‐response to ursodeoxycholic acid, as a first‐line therapy, is associated with a heightened risk of clinical events in primary biliary cholangitis (PBC). Herein, we determine whether biochemical non‐response to second‐line therapy in obeticholic acid (OCA) is also predictive of long‐term event‐free survival.

**Design:**

Data were collected from patients who initiated OCA at large, high‐volume centres in the UK, Italy, and Canada between August 2017 and 2019, with follow‐up continuing until June 2024. Biochemical non‐response was defined by POISE criteria. Clinical events were defined as hepatic decompensation, referral for transplantation, hepatocellular carcinoma, or death.

**Results:**

Our cohort consisted of 336 patients (29% with cirrhosis), of whom *n* = 150 (45%) discontinued OCA over 48 months. Over 851 patient‐years of OCA use, without the addition of another PBC therapy, *n* = 230, *n* = 192, *n* = 158 and *n* = 150 patients completed 12, 24, 36 and 48 months follow‐up, respectively. Of this cohort, 37%, 48%, 63% and 55% attained biochemical response, with 7%, 14%, 25% and 19% normalising ALP (*p* < 0.01; all comparisons vs. baseline). Over 4 years, 64 patients experienced a clinical event. Twelve‐month biochemical non‐response associated with a heightened risk of clinical events (hazard ratio [HR]: 4.50; 95% CI: 1.74–20.23), as did cirrhosis (HR: 20.24, 10.15–40.32), hyperbilirubinaemia (HR: 2.55, 1.71–3.76), hypoalbuminaemia (HR: 0.92, 0.90–0.96) and thrombocytopenia (HR: 0.99, 0.98–0.99). The prognostic utility of biochemical non‐response (HR: 3.29, 1.72–14.96) and cirrhosis (HR: 19.67, 5.09–76.08) persisted on multivariable analyses.

**Conclusion:**

Biochemical response stratifies risk of clinical events in PBC patients under OCA treatment. Whilst response rates increase over time, discontinuation rates underscore the need for newer treatment paradigms.

## Graphical abstract

In a multi‐centre study of obeticholic acid in primary biliary cholangitis, 45% discontinued the drug over 4 years, with 11% moving to combination therapy (fibrates). Of those continuing, response rates increased from 37% (1 year) to 55% (4 years). Non‐response at 12 months and cirrhosis were independent predictors of liver‐related events.

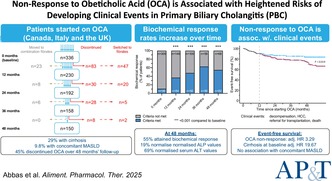

**Abbreviations**
ALP: alkaline phosphatase
ALT: alanine aminotransferase
AMA: anti‐mitochondrial antibody
AST: aspartate aminotransferase
HR: hazard ratio
IQR: inter‐quartile range
OCA: obeticholic acid
OR: odds ratio
PBC: primary biliary cholangitis
UDCA: ursodeoxycholic acid
ULN: upper limit of normal

## 1 Introduction

Primary biliary cholangitis (PBC) is a chronic, immune‐mediated disease characterised by bile duct inflammation, cholestasis and progressive liver fibrosis [1]. Despite the introduction of ursodeoxycholic acid (UDCA), clinical outcomes dwarf those of the background population, with up to 40% of patients developing cirrhosis [2]. Moreover, whilst data from historic UDCA trials demonstrate biochemical and histological improvements [3, 4, 5, 6, 7, 8, 9, 10, 11, 12], they do not show overt survival benefit. It was only through long‐term post hoc analyses, real‐world data and the advent of large multicentre registries that UDCA was seen to confer improved transplant‐free survival, resulting in widespread clinical adoption [13, 14, 15, 16, 17, 18].

However, not all UDCA‐treated patients respond to the drug equally. Broadly speaking, patients who lower serum liver biochemistry to pre‐specified thresholds (deemed biochemical responders) have a similar life expectancy to the general public [16, 17, 18, 19, 20]. Conversely, 30%–40% of patients inadequately respond to UDCA, with 5%–10% being drug intolerant [21]. These individuals are at an increased risk of liver disease progression and therefore candidates for second‐line therapy [22, 23, 24, 25, 26, 27].

In 2017, the synthetic farnesoid X receptor agonist, Obeticholic acid (OCA), gained market entry as a second‐line therapy for PBC. Robustly conducted controlled trials showed that OCA treatment was associated with a reduction in serum alkaline phosphatase (ALP) values among patients with inadequate response (or intolerance) to UDCA [28, 29, 30]. The long‐term extension studies that followed show that biochemical response rates to OCA are durable and sustained for up to 5 years on treatment [31, 32].

Regardless, market approval of OCA was initially granted on a conditional basis, with an ask that long‐term outcome data be provided through phase IV confirmatory clinical trials. Recruitment to such programmes has proven challenging, given the rare disease nature of PBC [33] and patients opting to start commercially available OCA rather than chance being randomised to the placebo arm of a clinical trial [34]. Consequently, a phase IV study of OCA was terminated early [34], and drug efficacy questioned by regulators [35]. This is despite the fact that patients under active treatment experience fewer clinical events when compared to real‐world, non‐OCA‐treated cohorts [32, 36].

In an attempt to overcome the hurdles associated with post‐market approval trials in a rare disease with a slow (albeit progressive) clinical course, we conducted a study of OCA treatment in a real‐world cohort of PBC patients. Our goal was to extrapolate from ‘lessons learned’ in studying UDCA; specifically, whether biochemical response under OCA treatment can also predict the development of future clinical events. An additional exploratory objective was to quantify the rates of switching OCA to off‐licence fibric acid derivatives (fenofibrate or bezafibrate), and how the rates of biochemical response on combination therapy (OCA and fenofibrate/bezafibrate) differ from second‐line regimens without OCA.

## 2 Patients and Methods

### 2.1 Study Cohort and Timelines

This was an international multicentre study, in which individual patient data was collected from the point of OCA initiation as a second‐line therapy in PBC. The index study date (equivalent to ‘first patient, first visit’) was August 2017; the point of market entry for OCA. Data was collected for all patients who commenced OCA in participating study sites from August 2017 to August 2019, with follow‐up continuing for a minimum of 4 years (until June 2024; equivalent to ‘last patient, last visit’).

In the UK, indications for starting second‐line therapy are in accordance with the National Institute for Health and Care Excellence (NICE) and British Society of Gastroenterology guidance (BSG); namely in patients with a persistently elevated ALP value ≥ 1.67× upper limit of normal (ULN) and/or an elevated bilirubin above the ULN despite 12 months of UDCA; or as OCA monotherapy in the event of UDCA intolerance [37]. Importantly, prescribing privileges for OCA in the UK are restricted to a finite number of high‐volume PBC centres, which serve as a network ‘hub’ for regional district general ‘spoke’ hospitals [38]. In our previous study, on‐treatment biochemical changes were presented over a 12‐month period, stemming from 14 UK networks [38]. All 14 networks were invited to participate in the longer‐term follow‐up programme presented herein. In addition, patients who were treated with OCA in Italy and Canada were also included. Notably, the indications for starting second‐line therapy differ in Italy, with ALP values of 1.5× ULN being the threshold to initiate treatment [39].

### 2.2 Study Definitions

Centres were asked to capture data prospectively, including patient demographics, treatment exposure (including the addition of, or switching to, off‐licensed fibric acid derivatives such as bezafibrate or fenofibrate); laboratory parameters (including, but not limited to, serum alanine aminotransferase [ALT], albumin, alkaline phosphatase [ALP], anti‐mitochondrial status [AMA], bilirubin, creatinine; circulating platelet count and peripheral blood haemoglobin values); treatment history with regard to UDCA; UK‐PBC scores [14]; transient elastography readings (where available, performed routinely, and with a minimum of 10 readings having > 70% success rate and an interquartile range < 30%); evidence of cirrhosis; and the incidence of clinical events. As liver biopsy is not routine standard of care, the presence of cirrhosis was determined non‐invasively through imaging features (coarse, irregular liver, splenomegaly, reversed portal vein flow, the presence of ascites), laboratory indices (using serum albumin values and circulating platelet counts) and available transient elastography readings, as described previously [38, 40].

Individuals were excluded from the study if follow‐up data were insufficient (< 2 clinic visits recorded), and in the event of confirmed past/concomitant hepatitis B virus (HBV) or hepatitis C virus (HCV) infection, Wilson disease, alpha‐1 antitrypsin deficiency, hereditary haemochromatosis, alcohol‐related liver disease, primary sclerosing cholangitis, or patients taking immunosuppressive therapy (for instance, due to concern of an overlap phenotype with autoimmune hepatitis). Individuals with a prior history of treatment with fibric acid derivatives, hepatic decompensation, or referral for transplant assessment/previously transplanted were also excluded.

Biochemical response was defined according to criteria set out in the registrational PBC OCA International Study of Efficacy trial (POISE); namely, an ALP value of < 1.67× the upper limit of normal (ULN) with a reduction of ≥ 15% from baseline and a normal total serum bilirubin [28]. Additionally, we determined the proportion of patients at 12, 24, 36 and 48 months who normalised serum ALT and ALP values. On‐treatment biochemical changes, drug discontinuation rates, and the proportion of patients completing follow‐up are presented at the same pre‐specified intervals, alongside those who stopped OCA and commenced fibric acid derivatives, or who commenced fibric acid derivatives in addition to OCA due to incomplete/non‐response or OCA intolerance.

Clinical events were defined as the development of hepatic decompensation (ascites, hepatic encephalopathy or variceal bleeding), hepatocellular carcinoma, referral for liver transplant assessment, or death from any cause.

### 2.3 Data Presentation and Statistical Analysis

Data are presented using median and interquartile ranges (IQR) for continuous variables. Liver enzymes (serum ALT and ALP values) are expressed as ratios to the upper limit of normal unless otherwise specified. The Wilcoxon signed‐rank test was applied to analyse differences in continuous variables from baseline to 12, 24, 36 and 48 months. Nominal data are presented as absolute values (percentages in parenthesis), and differences compared by Fisher's exact test. On‐treatment biochemical changes for the OCA‐treated cohort are presented initially for patients at the aforementioned timepoints, until the point of drug discontinuation, initiation of a fibric acid derivative, date of last clinic follow‐up, or point of liver transplantation or death.

Associations between measured covariates (baseline and on‐treatment) and event‐free survival were determined using Cox proportional hazards regression (hazard ratios [HR] and 95% CIs) and Kaplan–Meier survivorship estimates. In the event multiple liver‐related events were experienced, time to first event was taken in survivorship estimates. The association between baseline covariates and development of clinical events was determined akin to a ‘per‐protocol’ process, wherein any and every patient who received OCA contributed to the event‐free survival analysis, with censoring of follow‐up at the time of drug discontinuation, starting a fibric acid derivative, or entry into a clinical trial of a new investigational agent. Exploratory sub‐group analysis, as it relates to on‐treatment biochemical changes, rates of biochemical response and normalisation, and event‐free survival was also conducted for patients with (suspected) concomitant MASLD. As histological assessment is not routine standard of care in PBC, in the absence of liver biopsy, the presence of MASLD was defined by a controlled attenuation parameter (CAP) > 285 dB/m [41], and/or local investigator discretion in the presence of metabolic risk factors (including, but not limited to an elevated body mass index [BMI], diabetes mellitus, and hyperlipidaemia).

All analyses pertaining to rates (and reasons) of drug discontinuation, on‐treatment biochemical changes, and associations between biochemical response rates to OCA and future risks of clinical events were conducted for the pooled patient groups from the UK, Canada and Italy. Sub‐group analysis was then performed for the UK cohort as it relates to comparing the combination of OCA and fibric acid derivatives versus switching therapy (from OCA to fibric acid derivatives). As ongoing data capture following the initiation of fibric acid derivatives was an exploratory objective for the UK cohort specifically, comparisons between combination versus switching therapy groups were not possible from study centres in Italy and Canada.

### 2.4 Quality Control

Completeness, plausibility, and validity of data were carefully verified at source by referring centres, and again at the study coordinating centre (by investigators N.A. and P.J.T.). Where needed, individual site‐centre visits were conducted for UK centres, with an objective review of historical medical charts to retrieve missing data. This study was conducted in accordance with the Declaration of Helsinki. The protocol was reviewed and approved by the Institutional Research Board of the initiating centre (Birmingham; CARMS 14238), with individual participating hospital approval in accordance with local regulations.

### 2.5 Patient and Public Involvement

The study proposal was presented to the PBC Foundation (an international organisation committed to supporting people living with PBC) to obtain further perspective and comments, and to ensure findings can be translated and disseminated to the broader patient community. In collaboration, a lay abstract will be published and made available to the patient population through the periodical ‘PBC Bear Facts,’ along with a non‐technical summary of study results. Additionally, the longer‐term experiences of second‐line therapy will continue to be studied and presented, together with a full breakdown of potential side effects and details of putative drug‐induced hepatotoxicity.

## 3 Results

### 3.1 Characteristics of the Patient Cohorts

**FIGURE 1:**
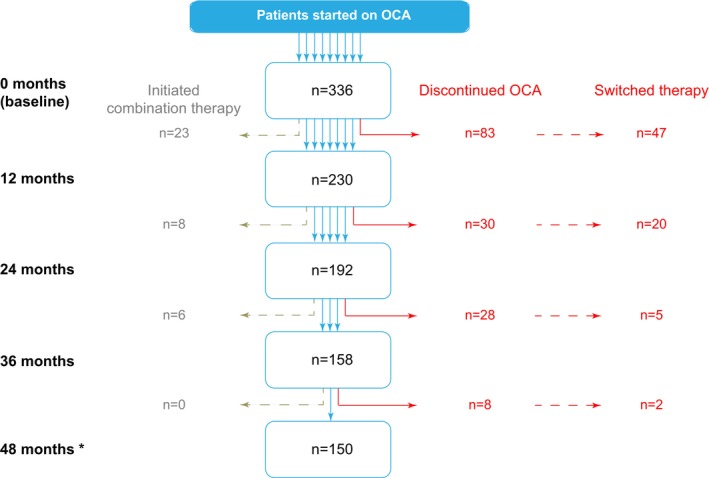
Study population. Between August 2017 and June 2024, data were accrued from a total of 336 individuals across sites in the UK, Italy and Canada. Indicative numbers of patients completing 12, 24, 36, and 48 months of follow‐up whilst taking obeticholic acid (OCA) in the absence of starting another second‐line agent are shown in black text, alongside those who initiated combination therapy with a fibric acid derivative in grey text, and individuals who discontinued OCA and initiated fibric acid derivatives as an alternative therapy in red text. * Seven patients initiated combination therapy after 48 months, in addition to those from earlier timepoints.

Data was captured from 336 individuals who initiated OCA as a second‐line treatment for PBC; 90% started treatment due to UDCA non‐response and the remainder due to UDCA intolerance. The majority of patients were women, with a median age of 53 years at treatment initiation, and 91% were UDCA‐treated (Table 1). Pruritus of any degree was reported in 44% of patients at baseline, with 29% of the overall cohort having cirrhosis. At 12–24, 36–48 months of follow‐up, *n* = 230 (68%), *n* = 192 (57%), *n* = 158 (47%) and *n* = 150 (45%) of patients, respectively, remained on OCA without the addition of, or switching to, a fibric acid derivative (Figure 1), which equated to 851‐patient‐years of medication use.

**TABLE 1:** Baseline Characteristics of the Study Population.

Age at PBC diagnosis	45 (39–51) years
Age at starting OCA	53 (49–61) years
Female sex	302 (89%)
AMA positive	301 (90%)
UDCA treated	309 (91%)
History of pruritus	148 (44%)
BMI	26 (23–29) kg/m^2^
Concomitant diabetes mellitus	14 (4%)
Concomitant MASLD	33 (9.8%)
Cirrhosis	96 (29%)
Portal hypertension	78 (23%)
Transient elastography reading *	9.5 (7.0–13.8) kPa
CAP	224 (186–268)
Starting doses of OCA	
5 mg once a day	264 (79%)
5 mg every other day	24 (7%)
5 mg once weekly	35 (10%)
5 mg twice weekly	13 (4%)
Laboratory values (continuous) **	
ALT	1.33 (0.93–2.04) ×ULN
ALP	2.69 (1.96–3.83) ×ULN
Bilirubin	0.62 (0.40–1.00) ×ULN 40 (IQR 37–44)
Albumin	40 (36–43) g/L
Platelet count	195,000 (160,000‐245,000)/mL
Creatinine	74 (60–94) micromol/L
Laboratory values (categorical)	
ALT > 1× ULN	217 (65%)
ALP > 1.67× ULN	300 (89%)
Bilirubin > 0.6× ULN	173 (52%)
Bilirubin > 1× ULN	83 (25%)
UK‐PBC risk scores	
5 year	1.9 (0.7–4.1) %
10 year	5.9 (2.5–13.0) %
15 year	10.2 (4.5–22.9) %

*Note:* Continuous data expressed as median (interquartile range) and categorical data as raw numbers (percentages).

Abbreviations: ALP, alkaline phosphatase; ALT, alanine aminotransferase; AMA, anti‐mitochondrial antibody; AST, aspartate aminotransferase; BMI, body mass index; CAP, continuous attenuation parameter; MASLD, metabolic dysfunction associated steatotic liver disease; OCA, obeticholic acid; PBC, primary biliary cholangitis; UDCA, ursodeoxycholic acid.

* Transient elastography readings available for *n* = 96/336 patients at baseline.

** Serum bilirubin, ALT and ALP values denote readings relative to the laboratory upper limit of normal.

### 3.2 Safety, Tolerability and Clinical Events

Over the 48‐month follow‐up period, OCA was discontinued in 150 patients. The most common reason stated was pruritus (*n* = 38) followed by hepatic decompensation (*n* = 36) and non‐pruritus associated drug intolerance (*n* = 22), although reasons for discontinuation differed depending on treatment duration (Table 2). In all, 64 patients experienced at least one clinical event over the 48‐month follow‐up period; encompassing hepatocellular carcinoma (*n* = 4), decompensation (*n* = 35; 28 ascites, 13 hepatic encephalopathy, 15 variceal bleeding), referrals for liver transplant assessment (*n* = 37), or death from any cause (*n* = 18). OCA was discontinued in 77% of patients experiencing a clinical event.

**TABLE 2:** Rates and rationale for OCA discontinuation (*n* = 150).

(A) Overall cohort	
Reason given	No. of patients
Biochemical non‐response	22
Pregnancy	2
Pruritus	38
Disease progression	18
Decompensation	36
Miscellaneous intolerances*	19
Non‐compliance	15

(B) By follow‐up time since starting OCA				
Reason for stoppage	0–12 months	12–24 months	24–36 Months	36–48 Months
Biochemical non‐response	1	18	1	2
Pregnancy	2	0	0	0
Pruritus	31	5	1	1
Disease progression	1	4	5	8
Decompensation	2	4	16	8
Miscellaneous*	16	9	0	0
Non‐compliance	12	2	0	1

* Include GI side effects (diarrhoea, cramps, nausea, vomiting, abdominal pain), light‐headedness, dizziness and headache.

In a sub‐group of patients without cirrhosis (*n* = 33/240), doses lower than 5 mg once a day were started due to a history of difficult‐to‐treat (or persistent albeit mild) pruritus. A total of 8/33 patients in this sub‐group discontinued OCA within 12 months of treatment initiation, with a further two, five, and four patients stopping therapy at 24, 36 and 48 months, respectively.

### 3.3 On Treatment Biochemical Changes in Serum Biochemistry

**FIGURE 2:**
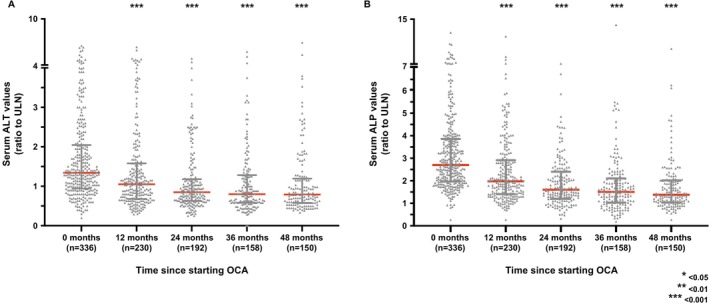
On‐treatment biochemical changes over time. Serum ALT (A) and ALP (B) values are presented for patients treated with obeticholic acid, who continued therapy at 12, 24, 36 and 48 months, in the absence of initiating a fibric acid derivative or other therapy for the treatment of primary biliary cholangitis. Values are expressed as a ratio to the upper limit of normal, with red lines indicating the median, and black whiskers indicating interquartile ranges. Asterisks indicate *p* values < 0.001 when comparing matched patient data at specific timepoints with readings taken at baseline (Wilcoxon signed‐rank test).

Observing the cohort in its entirety, median ALP values declined from 2.69× ULN at baseline (IQR 1.96–3.83) to 1.96× ULN at 12 months (IQR 1.40–2.90× ULN), 1.60× ULN at 24 months (IQR 1.20–2.39), 1.50× ULN at 36 months (IQR 1.03–2.09× ULN), and 1.38× ULN at 48 months (IQR 1.04–2.01× ULN) (*p* < 0.001 for all comparisons) (Figure 2). Accordingly, the median percentage reduction in serum ALP at 12, 24, 36 and 48 months was 27%, 41%, 44% and 55%, respectively. Significant changes in median ALT values were also observed across all time points, with reductions of 21%, 36%, 40% and 41% at 12, 24, 36 and 48 months, respectively.

**FIGURE 3:**
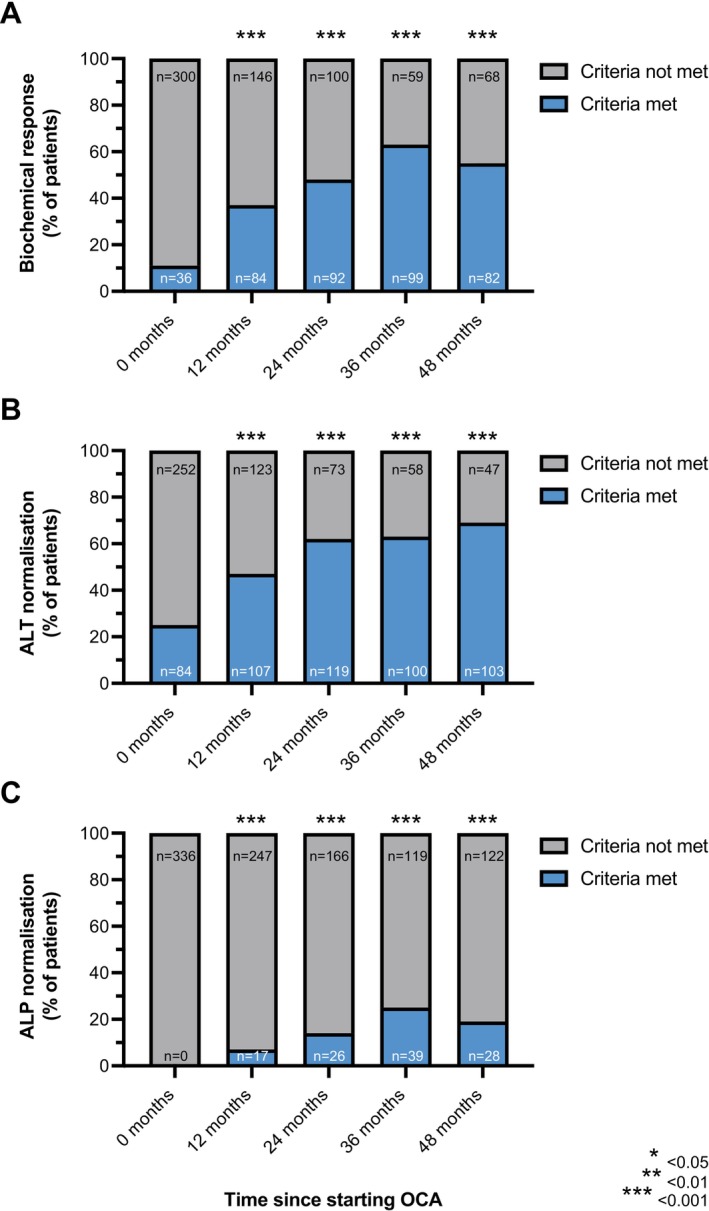
Rates of biochemical response and normalisation. The proportion of patients attaining biochemical response over time is shown according to the POISE criteria in (A), alongside normalisation rates in serum ALT (B) and ALP (C). Data shown for patients continuing OCA treatment in the absence of initiating a fibric acid derivative or other therapy for the treatment of primary biliary cholangitis. Asterisks indicate significant *p* values when comparing matched patient data at specific timepoints with readings taken at baseline (Fisher exact test).

No patient had normal serum ALP values at baseline, with rates of normalisation reaching 7% at 12 months, 14% at 24 months, 25% at 36 months and 19% at 48 months (*p* < 0.01 for all comparisons) (Figure 3). Under OCA therapy, the proportion of patients attaining complete normalisation in serum ALT increased over time from 25% at baseline to 47%, 62%, 63% and 69% at 12, 24, 36 and 48 months, respectively (*p* < 0.001; all comparisons).

Serum bilirubin values did not change significantly over time. However, on restricting analysis to patients with values above 0.6× ULN (a threshold associated with poorer clinical outcomes in PBC [42]), OCA therapy was associated with a reduction in bilirubin below this value in 58%, 61%, 54% and 59% of patients at 12, 24, 36 and 48 months (Figure S1).

Using the POISE criteria, 37%, 48%, 63% and 55% attained biochemical response at 12, 24, 36 and 48 months, respectively (Figure 3). The proportion of patients without portal hypertension who attained biochemical response mirrored those of the overall cohort, with rates of 35%, 53%, 66% and 63% at 12, 24, 36 and 48 months, respectively (Figure S2). Accordingly, rates of ALP normalisation and ALT normalisation were 7%, 14%, 26% and 23%, and 50%, 72%, 74% and 78%, respectively (Figure S2).

Of the non‐cirrhotic sub‐group who initiated OCA at low starting doses and continued treatment, daily dosing at a minimum of 5 mg once daily was attempted during follow‐up for all patients at varying intervals. On‐treatment biochemical response was met in *n* = 8/25 (32%), 11/23 (48%), 7/18 (39%) and 4/14 (26%) patients who continued OCA at 12, 24, 36 and 48 months, respectively.

### 3.4 Treatment Experience in Cirrhosis

In total, 96 patients had cirrhosis at baseline, of whom 91 were taking UDCA (Table S1). The starting doses of OCA at treatment initiation were 5 mg once a day in 42 patients, 5 mg every other day in 12 patients, 5 mg once weekly in 28 patients and 5 mg twice weekly in 8 patients. Seventy‐three patients (84%) manifest serum bilirubin values > 0.6× ULN, a threshold that stratifies the risk of disease progression in UDCA monotherapy cohorts [42, 43]. This was as compared to 100 patients without cirrhosis (45.9%); *p* < 0.001. Over time, the biochemical response criterion was met in 39%, 65%, 66% and 67% of patients in the cirrhotic sub‐group at 12, 24, 36 and 48 months (*p* < 0.05 when compared to baseline; all comparisons). However, *n* = 54/96 patients (56%) discontinued treatment within the 4‐year follow‐up period. The vast majority who discontinued OCA (*n* = 36/54) did so because of progressive liver disease, with 26 undergoing liver transplantation and 16 dying.

### 3.5 Predictors of Clinical Events Under OCA Therapy

**FIGURE 4:**
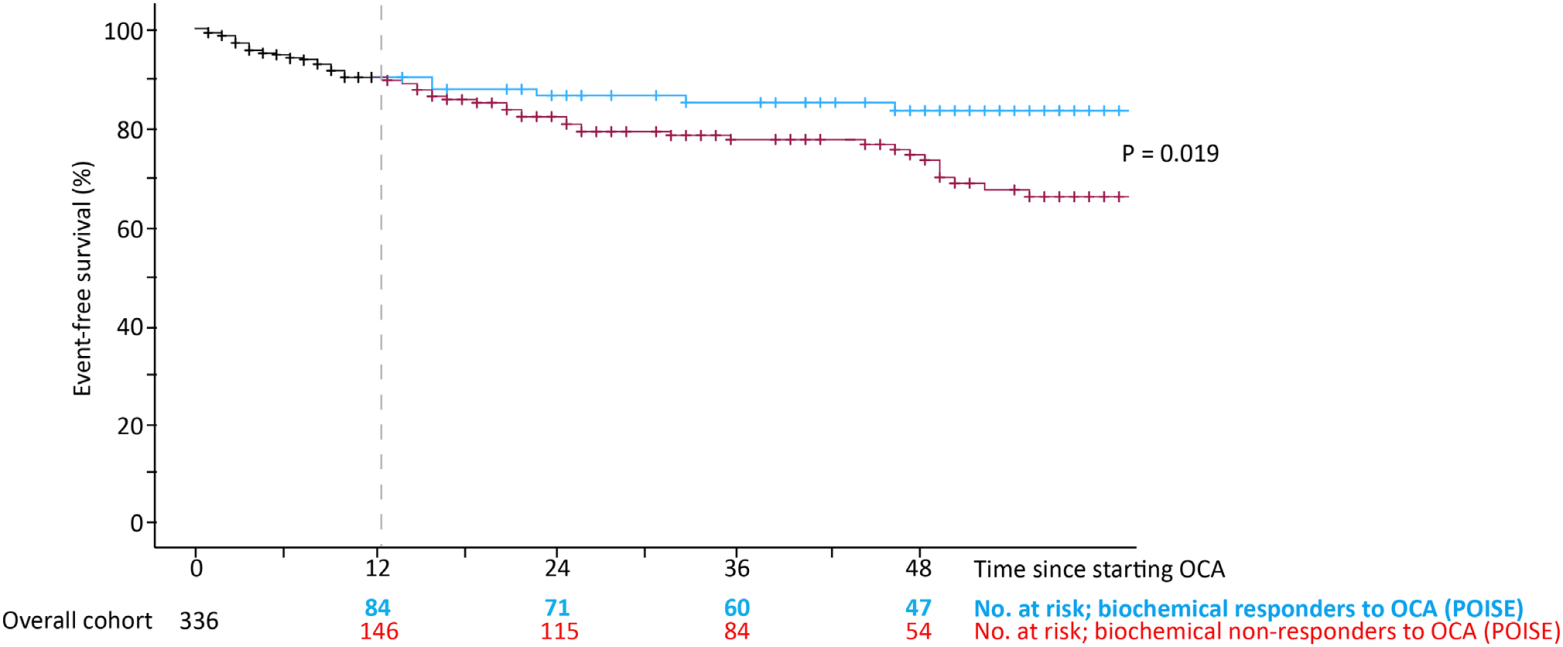
Event‐Free Survival Rates Under OCA Treatment. Kaplan–Meier survivorship estimates stratified according to one‐year biochemical response status under treatment with obeticholic acid. Analysis performed as time to index clinical event, defined as the first occurrence of hepatic decompensation, hepatocellular carcinoma, referral for liver transplantation, or death (any cause). On the occasion an event has not been met, censoring was performed at the date of last follow‐up, date of stopping obeticholic acid, or initiation of fibric acid therapy (whichever is first).

The group of patients who developed a clinical event trended to be of a younger age at the time of starting OCA compared to the overall cohort, with a larger proportion reporting pruritus at baseline (Table S2). On univariate analysis, factors associated with future risk of clinical events included having cirrhosis at baseline, portal hypertension at baseline, elevated baseline bilirubin, low serum albumin, lower circulating platelet count, and being a biochemical non‐responder to OCA after 1 year of therapy (Table 3; Figure 4). No significant differences in event‐free survival were observed among patients with versus without concomitant MASLD (Table 3).

**TABLE 3:** Predictors of clinical events.

	Unadjusted HR (95% CIs) CI)	*p* value	Adjusted HR (95% CIs) CI)	*p* value
Age at PBC diagnosis (per year increase)	1.00 (0.95–1.04)	0.96	—	—
Age at starting OCA (per year increases)	0.99 (0.97–1.02)	0.82	—	—
Female sex	2.33 (0.31–17.29)	0.40	—	—
History of pruritus at baseline	1.00 (0.96–1.05)	0.31	—	
AMA positivity	3.52 (0.45–27.32)	0.22	—	—
UDCA non‐treatment	1.96 (0.72–5.30)	0.01	n.s	—
BMI	1.00 (0.95–1.05)	0.92	—	—
Concomitant MASLD	0.58 (0.21–1.59)	0.29	—	—
Concomitant diabetes mellitus	2.35 (0.99–5.55)	0.052	n.s	—
Cirrhosis at baseline*	20.24 (10.15–40.32)	< 0.001	19.67 (5.09–76.08)	< 0.001
Portal hypertension at baseline**	2.03 (1.21–3.39)	0.01	—	—
Baseline laboratory values				
ALT (per unit increase above ULN)	1.20 (0.97–1.51)	0.11	n.s	—
ALP (per unit increase above ULN)	1.00 (1.00–1.01)	0.61	n.s	—
Bilirubin (per unit increase above ULN)	2.55 (1.71–3.76)	< 0.001	n.s	—
Albumin (per unit increase)	0.92 (0.90–0.96)	< 0.001	n.s	—
Platelet count (per unit increase)	0.99 (0.98–0.99)	< 0.001	n.s	—
Creatinine (per unit increase)	1.00 (1.00–1.01)	0.53	—	—
POISE non‐response at 12 months	4.50 (1.74–20.23)	0.01	3.29 (1.72–14.96)	0.012

*Note:* Data presented reflects the output of univariate (unadjusted HRs) and multivariable (adjusted HRs) Cox regression. Only variables with a *p* value < 0.1 on univariate analysis were entered into multivariable models.

Abbreviations: ALP, alkaline phosphatase; ALT, alanine aminotransferase; AMA, anti‐mitochondrial antibody; AST, aspartate aminotransferase; CAP, continuous attenuation parameter; CI, confidence interval; HR, hazard ratio; MASLD, metabolic dysfunction associated steatotic liver disease; OCA, obeticholic acid; PBC, primary biliary cholangitis; UDCA, ursodeoxycholic acid.

* Transient elastography readings were available for *n* = 96/336 patients at baseline. In this group of patients, the univariate HR for fibrosis readings (kPa) was 0.96, 95% CI 0.85–1.09, *p* = 0.52; and for CAP was univariate HR 1.00, 95% CI 0.99–1.01, *p* = 0.63.

** Additionally, the presence of portal hypertension at baseline was significant on univariate analysis (HR 2.02, 95% CI 1.21–3.19, *p* = 0.01). Given the interaction term with cirrhosis at baseline, only the latter was fed into the final multivariable model.

In a multivariable model, the presence of cirrhosis and biochemical non‐response to OCA at 1 year of therapy remained predictive of clinical events (Table 3; Figure S3). Moreover, the prognostic utility of biochemical non‐response at 1 year of OCA therapy (with regard to future risks of clinical events) was retained in the sub‐group without portal hypertension (Figure S4).

### 3.6 Experience of Combination OCA and Fibrate Therapy Compared to Fibrate Therapy Alone

Next, we performed exploratory sub‐analysis on the UK group of patients who initiated a fibric acid derivative during follow‐up. Specifically, we compared biochemical changes in the group who initiated treatment alongside OCA (combination therapy group) to those who discontinued OCA and switched to either bezafibrate or fenofibrate (switch therapy group). Of the 240 UK patients treated with OCA (median age 46 year; *n* = 48 with cirrhosis), 44 initiated a fibric acid derivative in the combination therapy group (*n* = 40 OCA with bezafibrate, and four patients OCA with fenofibrate), of whom 31 were also taking UDCA. In turn, 74 patients switched from OCA to a fibric acid derivative (*n* = 57 bezafibrate; *n* = 17 fenofibrate), of whom 61 were also taking UDCA. No significant differences were observed as relate to the baseline characteristics between combination and switch therapy groups (Table S3). Follow‐up at 1, 2, 3 and 4 years was completed by 29, 25, 21 and 22 patients in the combination group and by 57, 47, 38 and 37 patients in the switch therapy group. Reasons for OCA discontinuation in the switch therapy group were similar to those of the overall starting cohort (Table S4).

Biochemical response according to POISE criteria was met in 31%, 44%, 86% and 91% of pts. at 1, 2, 3 and 4 years., respectively, in the combination therapy group. This compared to 16%, 34%, 42% and 43% in the switch therapy group (Figure S5). Comparatively, ALP normalisation rates were 3% versus 0%, 4% versus 6%, 24% versus 13% and 55% versus 35%, at 1, 2, 3 and 4 years, respectively. A trend toward greater normalisation rates in all liver biochemical parameters was observed in the combination therapy group versus the switch therapy group: 0% versus 3% (1 year); 7% versus 3% (2 years); 13% versus 5% (3 years) and 35% versus 8% (4 years). Over time, 5 (11%) patients in the combination therapy group experienced a clinical event versus 4 (5%) in the switch therapy group (hazard ratio: 2.2; IQR 0.5–8.6).

## 4 Discussion

Herein, we present an internationally representative experience of OCA use in routine clinical practice. In so doing, we show that biochemical response criteria used to stratify high‐ versus low‐risk patients under UDCA treatment can now be applied to OCA‐treated cohorts as relates to predicting future risk of clinical events. Additionally, biochemical response rates to OCA increase over time, beyond the first year of therapy. However, > 40% of patients discontinue OCA within 4 years of treatment. This highlights an ongoing need for new PBC treatments, either in isolation [44, 45], or in combination with OCA [46, 47, 48]. On sub‐analyses of UK patients who initiated off‐label fibric acid derivatives alongside OCA, biochemical response rates exceeded 90% at 4 years, with more than half attaining normal ALP values. Additionally, in exploratory subgroup analysis, the group discontinuing OCA and switching to a fibric acid derivative exhibited lower biochemical response rates over time compared to those who continued OCA. These results indicate the potential synergistic benefits of combined peroxisome proliferator‐activated receptor (PPAR) and FXR agonism in cholestasis.

Shorter‐term, real‐world data pertaining to OCA in PBC have been presented previously, reflected through cohorts from Italy, Spain, Portugal and North America [36, 39, 49, 50, 51, 52]. These studies have been instrumental in validating results of registrational clinical trials, clearly demonstrating on‐treatment biochemical changes following 12 months of OCA therapy. Moreover, collaborative outputs from the CLEO‐AIGO and Italian PBC consortia identified potential variables associated with liver‐related clinical events, specifically among patients with cirrhosis treated with OCA over a 1‐year period [53]. These findings were recently validated by the ColHai registry from Spain, wherein lower serum albumin, lower circulating platelet count and biochemical non‐response to OCA were identified as predictors of hepatic decompensation in a cohort of 96 patients with cirrhosis secondary to PBC [54]. Our data add to this growing body of evidence, by validating the prognostic utility of biochemical response criteria in a large OCA‐treated cohort, independently of cirrhosis, and with clinical event capture for more than 4 years post treatment initiation.

In our previous study of 1‐year outcomes, we found that approximately one in 5 patients stopped OCA within 12 months of treatment initiation, with a similar rate observed for fibric acid derivatives. The latter is mirrored by a Dutch PBC experience, in which ~20% of patients stop bezafibrate in the first year of treatment [55]. However, reasons for discontinuation seemingly differ between drugs, with early OCA stoppages due to pruritus, compared to drug‐induced liver and/or kidney injury with bezafibrate [38]. By contrast, OCA discontinuations later in the clinical course are mostly due to disease progression in those with established, advanced PBC. Since market entry, regulatory restrictions were introduced when using OCA in cirrhosis. This is due to the emergence of adverse events when incorrect dosing was applied to patients with cirrhosis, clinically significant portal hypertension, and/or elevated serum bilirubin [56]. Taken together, it is the authors' view that OCA use be limited to patients with compensated liver disease, a normal platelet count and normal bilirubin values. Importantly, rates of OCA discontinuation mirror those reported for several licensed treatments used in other chronic conditions, including biologics to treat inflammatory bowel disease, oral anti‐glycaemic agents used to treat diabetes mellitus, statins to treat hypertension, and anti‐hypertensives used to treat high blood pressure [57, 58, 59, 60, 61, 62].

Despite long‐term OCA use, a large proportion of patients did not normalise ALP values; a desired therapeutic target particularly for individuals of younger age and those with advanced fibrosis [42, 63]. To this effect, combination therapy with OCA and a fibric acid derivative is increasingly utilised in PBC practice [46, 47]. Preliminary data from the Global PBC Group show that combination therapy is associated with threefold greater odds of normalising serum ALP values compared to either second‐line treatment alone [46]. Moreover, the addition of a fibric acid derivative to OCA was associated with a significant reduction in pruritus intensity. Contemporary data from Europe has validated these findings, although ~40% of patients experienced an adverse event over a median of 35 months treatment, with 18.8% stopping OCA [47]. Before our study, it was therefore not clear if switching from OCA to a fibric acid derivative would yield different outcomes from pursuing a combination approach. However, we show that continuing OCA (when tolerated) alongside a PPAR agonist may be more effective than switching between treatments [48].

The inability to recruit high‐risk PBC patients (with advanced disease) to phase IV studies of OCA meant that it has not been possible to validate drug efficacy in clinical trials. This led to the European Medicines Agency (EMA) recommending withdrawal of OCA in member nations of the European Union, despite the wealth of evidence from real‐world studies and target emulation trials demonstrating drug effectiveness and improved event‐free survival among OCA‐treated versus non‐treated patients [32, 34, 36]. Whilst the EMA decision does not affect practice in the UK and Canada, the approval of next‐generation PPAR agonists [44, 64, 65, 66] offers new opportunities for patients, including those previously or currently treated with OCA. Future studies from our group will continue to explore the effects of OCA in the real‐world setting, including combination treatment approaches with elafibranor and seladelpar, alongside other therapies that are on the horizon for PBC [22, 23, 67, 68, 69, 70, 71].

Of note, the UK ODN system ensures equitable access to newly licensed medicines for PBC patients, some variation in clinical practice is inevitable. This includes access to transplantation and the management of difficult‐to‐treat pruritus [72]. Moreover, the wider degree of heterogeneity in clinical care models between countries was not evaluated [73], highlighting a notable limitation of our study. This is particularly relevant to combination versus switch therapy comparisons, wherein data was only available in the UK cohort of patients. Moreover, the reasons for applying a switch versus combination therapy approach were not captured for all patients.

Assessment of pruritus as a driver of drug discontinuation was also patient‐ and clinician‐reported in line with routine standard of care, rather than through quantitative assessment tools applied in clinical trials [44, 45, 69, 70, 71]. To this effect, we call for contemporary and abbreviated outcome measures (for instance, the PBC‐10 and itch numerical rating scales) to be embedded in routine clinical practice, as a means of furthering real‐world outcome and natural history studies [74]. Future efforts may also benefit from testing whether the newly developed OCA score (a tool applied to predict on‐treatment biochemical responses) is able to stratify the risk of future clinical events [51], in parallel to validating the prognostic utility of POISE criteria beyond the 4‐year period studied herein. Further exploration of OCA in PBC will, in part, be dictated by regulatory decisions. With involvement throughout the process, we strongly advocate a patient‐centred approach to these efforts, offering credible insight into the subjective experience of second‐line therapies, beyond those captured in short‐lived clinical trial programmes.

## Author Contributions

**Nadir Abbas:** investigation, writing – original draft, methodology, validation, writing – review and editing, project administration, data curation, formal analysis, software. **Rachel Smith:** writing – review and editing, data curation, investigation. **Ellina Lytvyak:** writing – review and editing, data curation, investigation, validation. **Miki Scaravaglio:** writing – review and editing, data curation, investigation, validation. **Neil Halliday:** writing – review and editing, data curation, investigation. **Amal Almahroos:** writing – review and editing, data curation, investigation. **Nadia Eden:** writing – review and editing, data curation, investigation. **Diane Lloyd‐Madden:** investigation, writing – review and editing, data curation. **Sanchit Sharma:** investigation, writing – review and editing, data curation. **James Ferguson:** investigation, writing – review and editing, data curation. **Jessica K. Dyson:** investigation, writing – review and editing, data curation. **Douglas Thorburn:** investigation, writing – review and editing, data curation. **David Jones:** writing – review and editing, data curation, investigation. **Aldo J. Montano‐Loza:** investigation, writing – review and editing, data curation, validation. **Marco Carbone:** investigation, writing – review and editing, data curation, validation. **Pietro Invernizzi:** investigation, writing – review and editing, data curation, validation. **George Mells:** writing – review and editing, data curation, investigation. **Emma L. Culver:** investigation, writing – review and editing, data curation. **Palak J. Trivedi:** funding acquisition, investigation, supervision, writing – review and editing, methodology, visualization, project administration, conceptualization, resources, writing – original draft.

## Conflicts of Interest

Nadir Abbas has received institutional salary support from the National Institute for Health and Social Care Research (NIHR), Biomedical Research Centre (BRC) and speaker and consulting fees from Advanz Pharma, Rocket medical, Signant health, Umecerine pharmaceuticals and Dr. Falk Pharma and grant support from Guts UK. Palak J. Trivedi receives institutional salary support from the National Institute for Health Research (NIHR) Birmingham Biomedical Research Centre (BRC). The views expressed are those of the author(s) and not necessarily those of the National Health Service, the NIHR, or the Department of Health. Prof. Trivedi has received grant support from the Wellcome Trust, the Medical Research Foundation, Guts UK, PSC Support, Innovate UK, LifeArc, NIHR, Advanz/Intercept Pharma, Dr. Falk Pharma, Gilead sciences, GlaxoSmithKline (GSK), Regeneron, Ipsen, Mirum Pharma, and Bristol Myers Squibb. He has also received speaker fees from Albireo/Ipsen, Advanz/Intercept, Mirum and Dr. Falk Pharma. He has received advisory board/consultancy fees from Cymabay/Gilead sciences, Advanz/Intercept, Albireo/Ipsen, Mirum, Dr. Falk Pharma and GSK.

## Supporting information


**Table S1:** Demographics of the study Cohort according to the presence of cirrhosis.
**Table S2:** Characteristics of patients experiencing clinical events (*n* = 64).
**Table S3:** Baseline characteristics of patients initiating fibric acid derivatives.
**Table S4:** Rates and rationale for OCA discontinuation in switch therapy group.
**Figure S1:** On‐treatment changes in serum bilirubin.
**Figure S2:** Rates of biochemical response and normalisation in the absence of portal hypertension.
**Figure S3:** Event‐free survival under OCA treatment stratified by biochemical response and presence of cirrhosis.
**Figure S4:** Event‐free survival stratified by biochemical response in the absence of portal hypertension.
**Figure S5:** Biochemical response rates under ‘combination’ compared to ‘switch’ therapy.

## Data Availability

The data that support the findings of this study are available from the corresponding author upon reasonable request.
