# Supplementary material for: Non‐Response to Obeticholic Acid Is Associated With Heightened Risks of Developing Clinical Events in Primary Biliary Cholangitis
Source: Aliment Pharmacol Ther. 2025 Sep 22;63(4):494–506. doi: 10.1111/apt.70378 (PMC12854645; doi:10.1111/apt.70378)
Supplement: Supplementary file 1 — Table S1: Demographics of the study Cohort according to the presence of cirrhosis. Table S2: Characteristics of patients experiencing clinical events (n = 64). Table S3: Baseline characteristics of patients initiating fibric acid derivatives. Table S4: Rates and rationale for OCA discontinuation in switch therapy group. Figure S1: On‐treatment changes in serum bilirubin. Figure S2: Rates of biochemical response and normalisation in the absence of portal hypertension. Figure S3: Event‐free survival under OCA treatment stratified by biochemical response and presence of cirrhosis. Figure S4: Event‐free survival stratified by biochemical response in the absence of portal hypertension. Figure S5: Biochemical response rates under ‘combination’ compared to ‘switch’ therapy. [file APT-63-494-s001.docx]

| Supplementary Table 1: Demographics of the Study Cohort according to the Presence of Cirrhosis | | | | |
| --- | --- | --- | --- | --- |
|  | **Cirrhosis (n=96)** | **No cirrhosis (n=240)** | **P value** |  |
| Age at PBC diagnosis | 46 (39-54) years | 47 (41-64) years | 0.38 |  |
| Age at starting OCA | 53 (49-61) years | 52 (48-57) years | 0.54 |  |
| Female sex | 86 (91%) | 216 (90%) | 0.46 |  |
| Starting dose of OCA  -5mg once a day  -5mg every other day  -5mg once weekly  -5mg twice weekly | 65 (68.4%)  8 (8.4%)  20 (21.1%)  2 (2.1%) | 203 (86%)  17 (7.2%)  12 (5.1%)  4 (1.7%) |  |  |
| UDCA treated | 91 (95%) | 222 (93%) | 0.56 |  |
| History of pruritus | 57 (60%) | 96 (40%) | 0.24 |  |
| Transient elastography reading | 22.2 (18.6-29.91) kPa | 8.4 (8.1-10.2) kPa | <0.001 |  |
| Portal hypertension | 33 (34%) | 45 (19%) | 0.002 |  |
| Laboratory values (continuous) * |  |  |  |  |
| -ALT | 1.38 (0.81-2.34) xULN | 1.35 (1.03-2.03) xULN | 0.87 |  |
| -ALP | 2.53 (1.79-3.62) xULN | 2.75 (2.04-3.96) xULN | 0.021 |  |
| -Bilirubin | 0.95 (0.76-1.93) xULN | 0.48 (0.33-0.67) xULN | <0.001 |  |
| -Albumin | 39 (36-43) g/L | 41 (39-46) g/L | 0.03 |  |
| -Platelet count | 161,000 (109,000-245,000) / mL | 190,000 (168,000-210,000) / mL | 0.001 |  |
| -Creatinine | 80 (64-120) micromol / L | 80 (65-96) micromol / L | 0.34 |  |
| Laboratory values (categorical) |  |  |  |  |
| -ALT >1xULN | 59 (66%) | 158 (74%) | 0.85 |  |
| -ALP >1.67xULN | 75 (83%) | 199 (91%) | 0.07 |  |
| -Bilirubin >0.6xULN | 73 (84%) | 100 (46%) | <0.001 |  |
| -Bilirubin >1xULN | 45 (53%) | 38 (17%) | <0.001 |  |
| UK-PBC risk scores |  |  |  |  |
| -5 year | 9.95% (4.45%-13.45%) | 2.7% (1.55%-3.5%) | <0.001 |  |
| -10 year | 29.59% (17.5%-43.5%) | 8.6% (4.6%-11.45%) | <0.001 |  |
| -15 year | 47.95% (28.5-65.5%) | 15.4% (12.4%-18.8%) | <0.001 |  |
| Continuous data expressed as median (interquartile range) and categorical data as raw numbers (percentages).  * Serum bilirubin, ALT and ALP values denote readings relative to the laboratory upper limit of normal.  Abbreviations: ALT, alanine aminotransferase; AST, aspartate aminotransferase; AMA, anti-mitochondrial antibody; ALP, alkaline phosphatase; OCA, obeticholic acid; PBC, primary biliary cholangitis; UDCA, ursodeoxycholic acid. | | | |  |

| Supplementary Table 2: Characteristics of Patients Experiencing Clinical Events (n=64) | |
| --- | --- |
| Age at PBC diagnosis | 44 (35-52) years |
| Age at starting OCA | 48 (36-54) years |
| Female sex | 59 (92%) |
| AMA positive | 62 (97%) |
| UDCA treated | 57 (90%) |
| History of pruritus | 41 (64%) |
| Cirrhosis at baseline | 51 (80%) |
| Portal hypertension | 23 (36%) |
| Transient elastography reading | 18.6 (11.6-24.8) kPa |
| Starting doses of OCA |  |
| -5mg once a day | 32 (50%) |
| -5mg every other day | 10 (14%) |
| -5mg once weekly | 22 (36%) |
| Laboratory values (continuous) * |  |
| -ALT | 1.65 (1.35-2.56) xULN |
| -ALP | 3.24 (2.31-4.59) xULN |
| -Bilirubin | 1.25 (0.65-2.34) xULN  40 (IQR 37-44) |
| -Albumin | 34 (31-36) g/L |
| -Platelet count  #  - | 145,000 (120,000-190,000) / mL  98 (980 |
| -Creatinine | 98 (80-116) micromol / L |
| Laboratory values (categorical) |  |
| -ALT >1x ULN  -AST >1xULN | 45 (70%)  164 (73%) |
| -ALP >1.67x ULN | 59 (93%) |
| -Bilirubin >1x ULN | 40 (62% |
| -Bilirubin >0.6x ULN | 55 (86%) |
| UK-PBC risk scores |  |
| -5 year | 18.4% (12.4%-24.5%) |
| -10 year | 45.4% (26.5%-57.5% |
| -15 year | 69.1% (45.3%-81.4%) |
| Continuous data expressed as median (interquartile range) and categorical data as raw numbers (percentages).  * Serum bilirubin, ALT and ALP values denote readings relative to the laboratory upper limit of normal.  Abbreviations: ALT, alanine aminotransferase; AST, aspartate aminotransferase; AMA, anti-mitochondrial antibody; ALP, alkaline phosphatase; OCA, obeticholic acid; PBC, primary biliary cholangitis; UDCA, ursodeoxycholic acid. | |

| Supplementary Table 3: Baseline Characteristics of Patients Initiating Fibric Acid Derivatives | | | | |  |
| --- | --- | --- | --- | --- | --- |
|  | | Combination OCA therapy (n=44) | Switch from OCA therapy (n=74) | P-value |  |
| Age at PBC diagnosis | | 44 (39-51) | 43 (40-53) | 0.41 |  |
| Age at starting OCA | | 54 (42-63) | 53 (44-62) | 0.23 |  |
| Age at starting fibric acid derivatives | | 55 (41-64) | 54 (41-60) | 0.38 |  |
| Female Sex | | 42 (95.5%) | 68 (94.4%) | 0.81 |  |
| AMA positive | | 39 (88.6%) | 65 (94.2%) | 0.28 |  |
| UDCA treated | 40 (90.9%) | 65 (87.8%) | 0.91 |  |  |
| History of pruritus | 22 (56.4%) | 35 (54.7%) | 0.86 |  |  |
| Cirrhosis at baseline | 7 (15.9%) | 12 (16.2%) | 0.91 |  |  |
| Laboratory values (continuous) *** |  |  |  |  |  |
| -ALT | 1.33 (0.93-2.04) xULN | 1.29 (0.87-2.14) xULN | 0.23 |  |  |
| -ALP | 2.61 (2.10-4.30) xULN | 3.01 (2.18-3.99) xULN | 0.18 |  |  |
| -Bilirubin | 0.56 (0.34-0.70) xULN  40 (IQR 37-44) | 0.60 (0.40-0.80) xULN | 0.34 |  |  |
| -Albumin | 43 (37-42) g/L | 42 (39-45) g/L | 0.21 |  |  |
| -Platelet count | 195,000 (160,00-245,000) / mL | 210,000 (170,000-260,000) / mL | 0.81 |  |  |
| -Creatinine | 74 (60-94) micromol / L | 84 (70-94) micromol / L | 0.64 |  |  |
| Laboratory values (categorical) |  |  |  |  |  |
| -ALT >1 xULN  -AST >1xULN | 19 (47.5%) | 40 (58%) | 0.29 |  |  |
| -ALP >1.67 xULN | 38 (92.7%) | 64 (92.8%) | 0.98 |  |  |
| -Bilirubin >0.6 xULN | 8 (20%) | 15 (21.7%) | 0.83 |  |  |
| -Bilirubin >1 xULN | 9 (13%) | 5 (12.5%) | 0.93 |  |  |
| Biochemical non-responders (POISE) | 38 (92.7%) | 64 (92.8%) | 0.989 |  |  |
| UK PBC risk scores |  |  |  |  |  |
| -5 year | 3.9% (1.6%-6.5%) | 4.5% (1.6%-6.9%) | 0.43 |  |  |
| -10 year | 6.5% (4.5%-8.5%) | 7.1% (5.4%-10.7) | 0.37 |  |  |
| -15 year | 15.3% (11.6%-19.5%) | 17.4% (11.6%-21.5%) | 0.21 |  |  |
| Continuous data expressed as median (interquartile range) and categorical data as raw numbers (percentages).  * Serum ALT, bilirubin and ALP values denote readings relative to the laboratory upper limit of normal.  Abbreviations: ALT, alanine aminotransferase; AST, aspartate aminotransferase; AMA, anti-mitochondrial antibody; ALP, alkaline phosphatase; OCA, obeticholic acid; PBC, primary biliary cholangitis; UDCA, ursodeoxycholic acid. | | | | | |

| **Supplementary Table 4:**  **Rates and Rationale for OCA Discontinuation in Switch Therapy Group** | |
| --- | --- |
| **Reason given** | **No. of patients** |
| Biochemical non-response  (prior to combination therapy era) | 26 |
| Pruritus | 16 |
| Disease progression | 2 |
| Decompensation | 0 |
| Miscellaneous intolerances* | 29 |
| Non-compliance | 1 |
| *Include GI side effects (diarrhoea, cramps, nausea, vomiting, abdominal pain), light-headedness, dizziness and headache | |

| **Supplementary Figure 1: On-treatment changes in serum bilirubin** |
| --- |
| 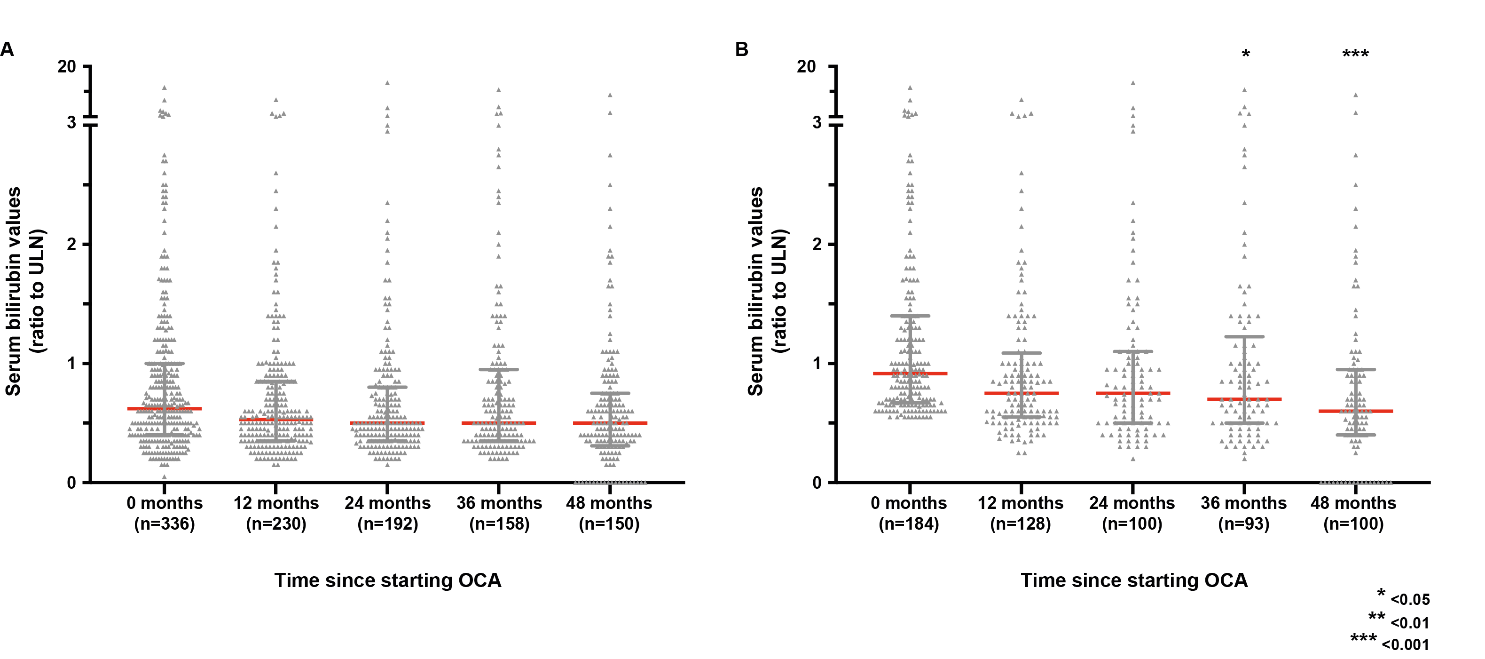 |
| Serum bilirubin values are presented for patients treated with obeticholic acid, who continued therapy at 12, 24, 36 and 48 months, in the absence of initiating a fibric acid derivative or other therapy for the treatment of primary biliary cholangitis. Data is shown for the overall cohort **(A)** and for the sub-group with baseline values above 0.6x the upper limit of normal **(B)**. Values expressed as a ratio to the upper limit of normal, with red lines indicating the median, and black whiskers indicating interquartile ranges. Asterisks indicate significant P values when comparing matched patient data at specific timepoints with readings taken at baseline (Wilcoxon signed-rank test). |

| **Supplementary Figure 2:**  **Rates of Biochemical Response and Normalisation in the Absence of Portal Hypertension.** |
| --- |
| **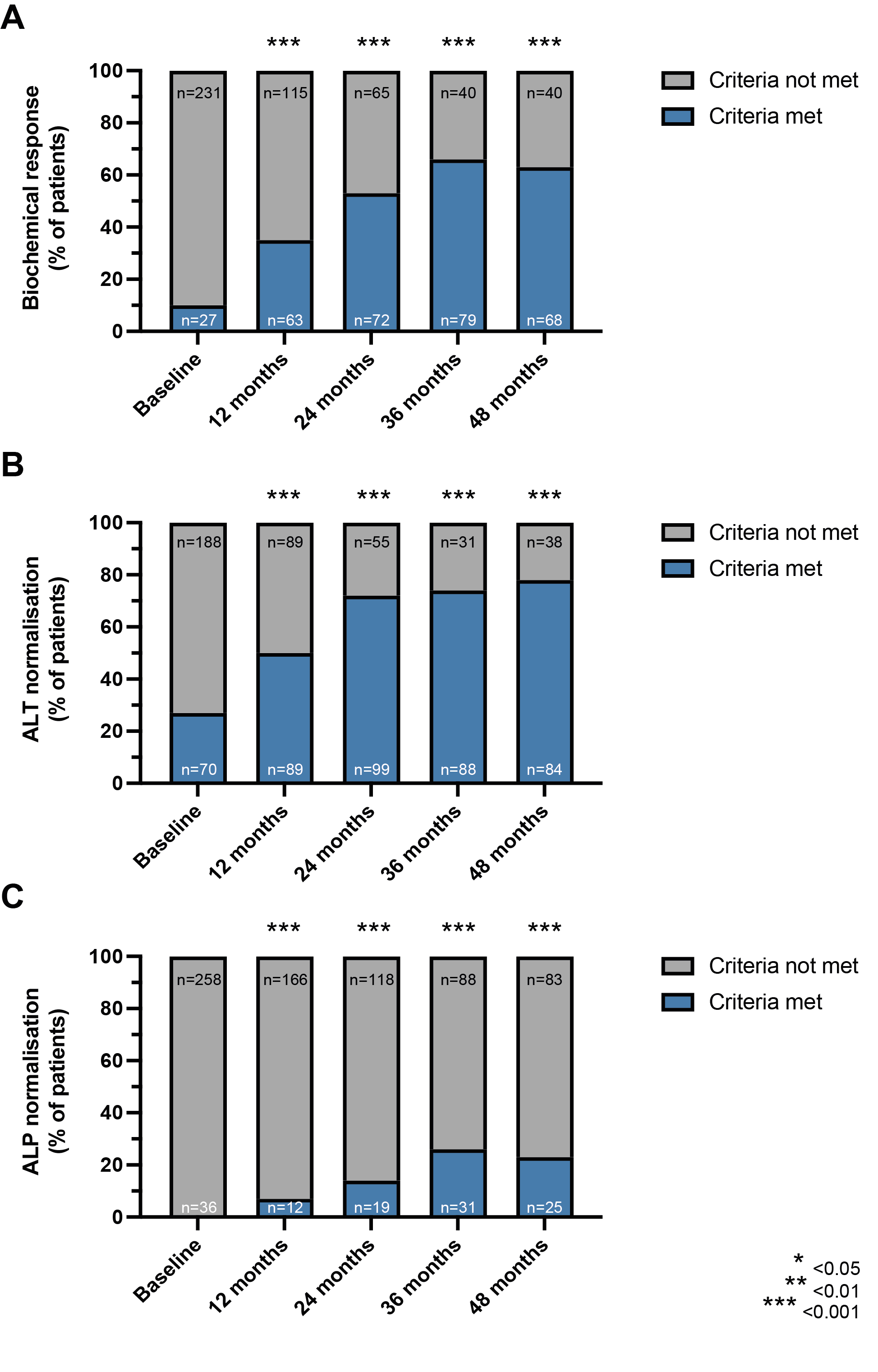** |
| The proportion of the non-portal hyertensive sub-group attaining biochemical response over time is shown according to the POISE criteria in (A), alongside normalisation rates in serum ALT (B) and ALP (C). Data shown for patients continuing OCA treatment in the absence of initiating a fibric acid derivative or other therapy for the treatment of primary biliary cholangitis. Asterisks indicate significant P values when comparing matched patient data at specific timepoints with readings taken at baseline (Fisher exact test). |

| **Supplementary Figure 3:**  **Event-Free Survival Under OCA Treatment Stratified by Biochemical Response and Presence of Cirrhosis** |
| --- |
| **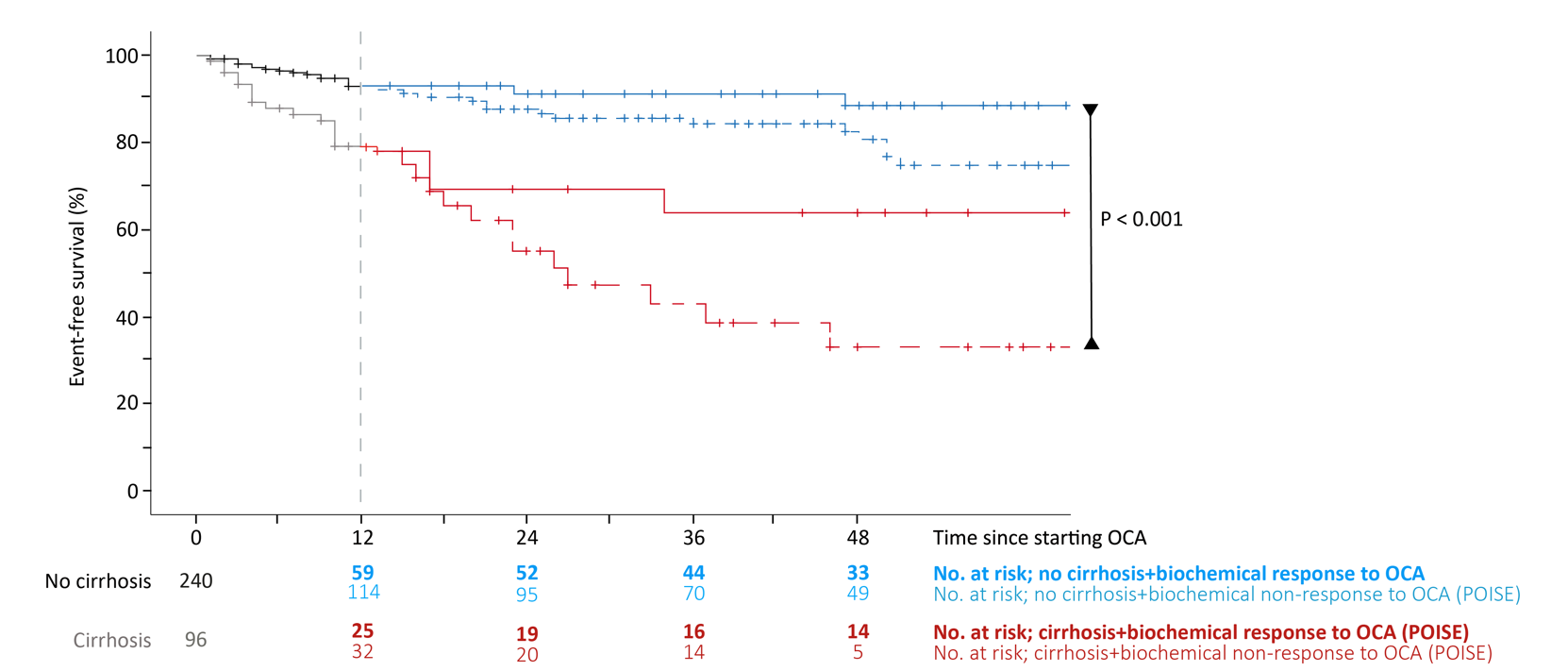** |
| Kaplan-Meier survivorship estimates stratified according to one-year biochemical response status under treatment with obeticholic acid and the presence of cirrhosis. Analysis performed as time to index clinical event, defined as the first occurrence of hepatic decompensation, hepatocellular carcinoma, referral for liver transplantation or death (any cause). On the occasion an event has not been met, censoring was performed at the date of last follow-up, date of stopping obeticholic acid, or initiation of fibric acid therapy (whichever is first). |

| **Revised Supplementary Figure 4:**  **Event-Free Survival Stratified by Biochemical Response in the Absence of Portal Hypertension** |
| --- |
| 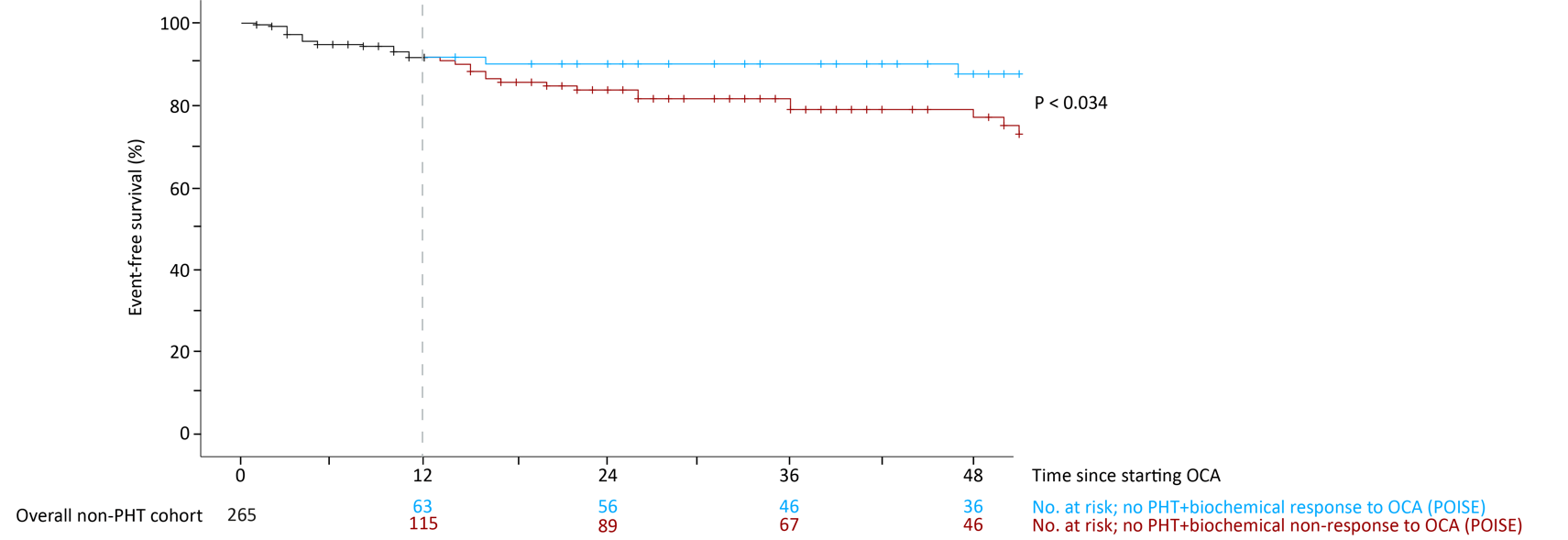 |
| Kaplan-Meier survivorship estimates stratified according to one-year biochemical response status under treatment with obeticholic acid, in the sub-group of patients without portal hypertension (PHT). Analysis performed as time to index clinical event, defined as the first occurrence of hepatic decompensation, hepatocellular carcinoma, referral for liver transplantation or death (any cause). On the occasion an event has not been met, censoring was performed at the date of last follow-up, date of stopping obeticholic acid, or initiation of fibric acid therapy (whichever is first). |

| **Supplementary Figure 5:**  **Biochemical Response Rates Under ‘Combination’ Compared to ‘Switch’ Therapy** |
| --- |
| 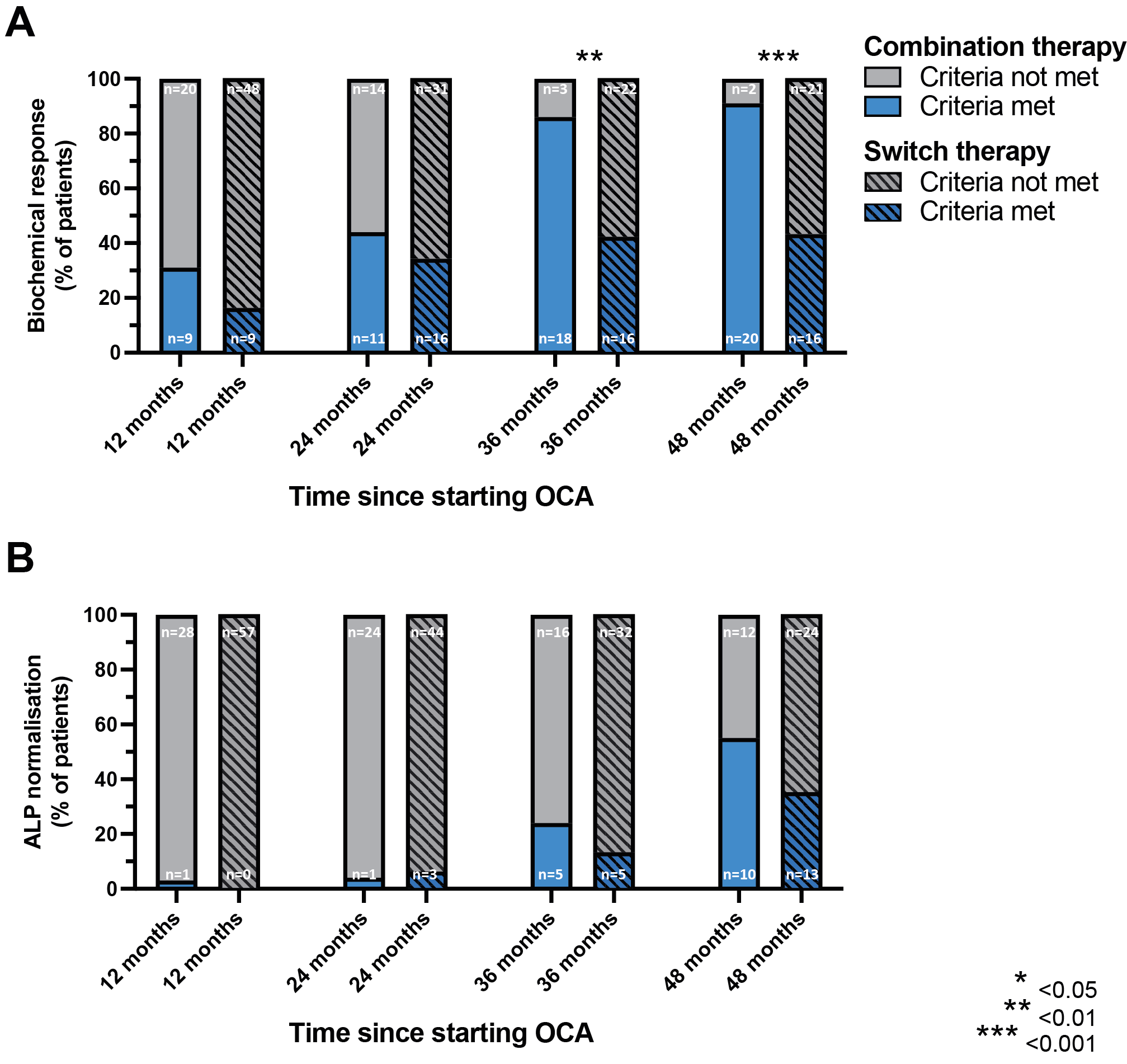 |
| The proportion of patients attaining biochemical response over time is shown according to the POISE criteria in (**A**), alongside normalisation rates in serum ALP **(B)**. Data shown for patients in the combination obeticholic (OCA) acid and fibric acid derivative group (plain boxes) and the group that switched from OCA to a fibric acid derivative (hashed boxes). Asterisks indicate significant P values when comparing matched patient data at specific timepoints with readings taken at baseline (Fisher exact test). |
